# Catalytic Alkyne Allylboration: A Quest for Selectivity

**DOI:** 10.1021/acscatal.3c03015

**Published:** 2023-09-13

**Authors:** Andrea Chaves-Pouso, Eva Rivera-Chao, Martín Fañanás-Mastral

**Affiliations:** † Centro Singular de Investigación en Química Biolóxica e Materiais Moleculares (CiQUS), Universidade de Santiago de Compostela, 15782 Santiago de Compostela, Spain

**Keywords:** copper, palladium, boron, alkynes, carboboration, allylic substitution, asymmetric catalysis

## Abstract

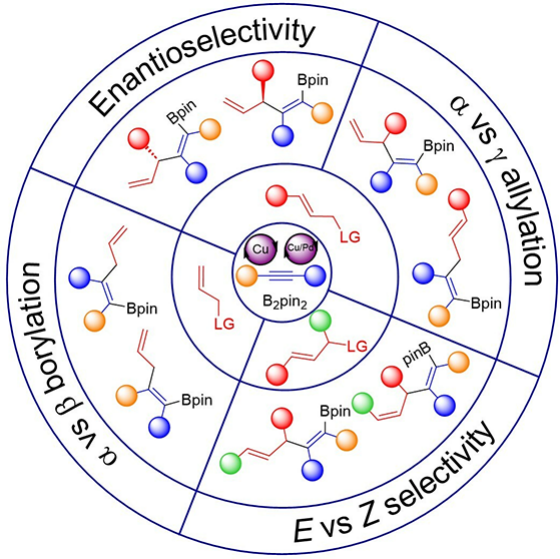

Catalytic methodologies that enable the synthesis of complex organic molecules from simple and readily available starting materials represent a goal in modern synthetic chemistry. In particular, multicomponent carboboration reactions that provide stereoselective access to densely functionalized building blocks are particularly valuable to achieve molecular diversity. This Perspective covers the developments in the area of catalytic allylboration of alkynes and highlights the key features that have allowed for the control of the regio-, diastereo-, and enantioselectivity in these transformations.

## Introduction

Catalytic allylic substitution through which an allylic electrophile and a nucleophilic reagent are converted to a product featuring a newly formed olefin represents a powerful tool in organic synthesis.^1^ While the majority of allylic substitution reactions still correspond to the incorporation of an alkyl nucleophilic fragment, significant advances have been made in the development of coupling reactions involving the addition of alkenyl reagents (i.e., allylic alkenylation). This transformation is particularly important since it gives straightforward access to the 1,4-diene unit, the so-called skipped diene, a prominent structural motif in a wide range of bioactive compounds and natural products.^2^ Traditionally, allylic alkenylation reactions have relied on the use of a stoichiometric amount of an alkenylmetal reagent using palladium,^3^ copper,^4^ iridium,^5^ and rhodium^6^ catalysis. Despite their efficiency, these protocols require the a priori preparation of the alkenylmetal reagent, thus adding an extra step to the method. Moreover, in some cases, their relatively high reactivity can compromise the functional group tolerance of the reaction.

**Scheme 1:**
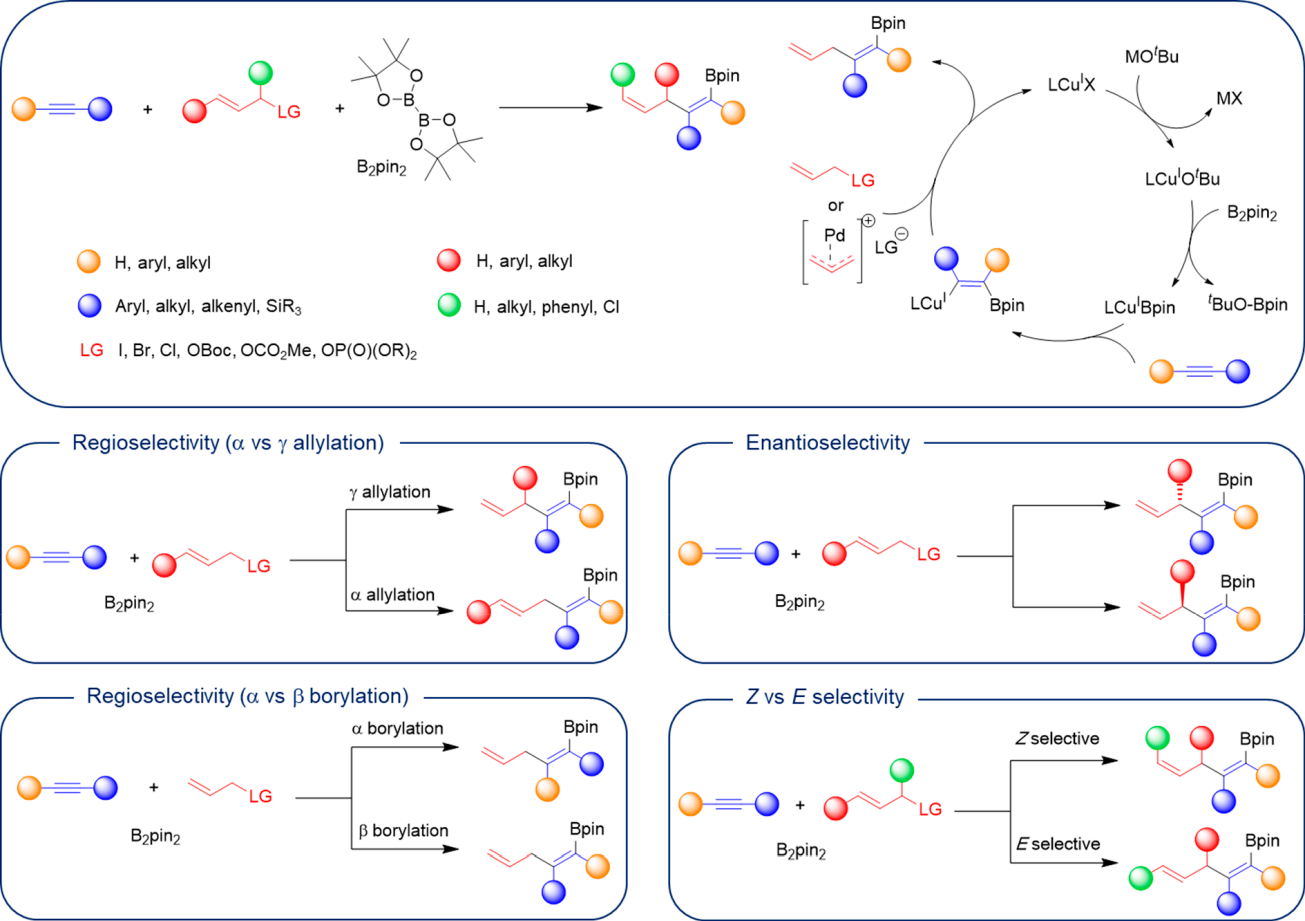
Allylboration of Alkynes: Mechanism and Selectivity Challenges

An interesting alternative to the stoichiometric use of organometallic reagents is the use of simple and abundant unsaturated hydrocarbons as pro-nucleophiles through their catalytic transformation into a transient organocopper species by reaction with a hydrosilane^7^ (or dihydrogen^8^), a silylborane,^9^ or a diboron compound.^10^ Trapping of the corresponding organocopper intermediate with a suitable reagent results in a reductive, silylative, or borylative coupling, respectively. In this context, alkynes have been described to react with boryl copper complexes through the insertion of the C–C triple bond into the Cu–B bond to afford a *syn*-stereodefined β-boryl-substituted alkenyl copper complex.^11^ On the basis of this strategy, several research groups have investigated catalytic transformations in which the transient alkenyl-Cu intermediate is trapped by an electrophile.^10^ If the electrophile is an allylic substrate, the process results in an allylic alkenylation involving the formation of a new C–C bond and a synthetically versatile C–B bond, i.e., a net allylboration reaction (Scheme 1). These transformations usually involve the catalytic formation of a copper alkoxide that reacts with a diboron compound (typically B_2_pin_2_) to generate a boryl copper complex that subsequently adds to the alkyne to afford the β-boryl-substituted alkenyl copper species, which reacts with the allylic compound, either directly or with the help of a cocatalyst, generally a Pd complex that synergistically activates it.^12^ Such a transformation entails an important selectivity challenge, as in addition to the common α-vs γ-allylation selectivity issue associated with the allylic substitution step, control over the chemoselective addition of the boryl-copper complex to the alkyne (vs the direct borylation of the allylic substrate) and regioselectivity of the alkyne borylcupration must also be addressed. The picture gets even more complicated when the reaction also demands control over the enantioselective formation of a new stereogenic center and/or over the stereoselective formation of a 1,2-disubstituted olefin arising from the use of a secondary allylic substrate (Scheme 1). This Perspective provides an overview of the catalytic alkyne allylboration with a special focus to highlight how copper catalysis or copper/palladium cooperative catalysis has been used to achieve control over all these selectivity points.

## Regioselectivity: α- versus γ-Allylation

**Scheme 2:**
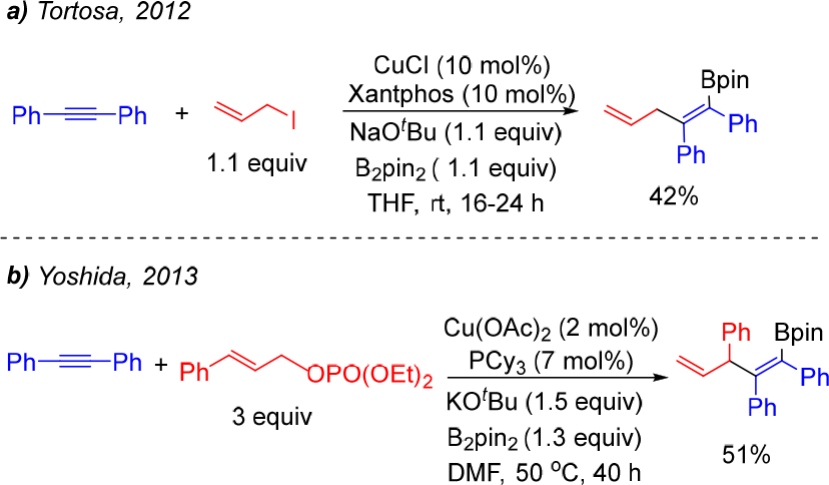
Early Examples of Copper-Catalyzed Allylboration of Alkynes

Preliminary results on alkyne allylboration were initially reported by Tortosa et al.^13^ and Yoshida et al.^14^ through single examples involving the copper-catalyzed coupling of diphenylacetylene and B_2_pin_2_ with simple allyl iodide and cinnamyl phosphate, respectively (Scheme 2). The latter was shown to proceed with perfect γ-allylation selectivity under Cu/PCy_3_ catalysis.

**Scheme 3:**
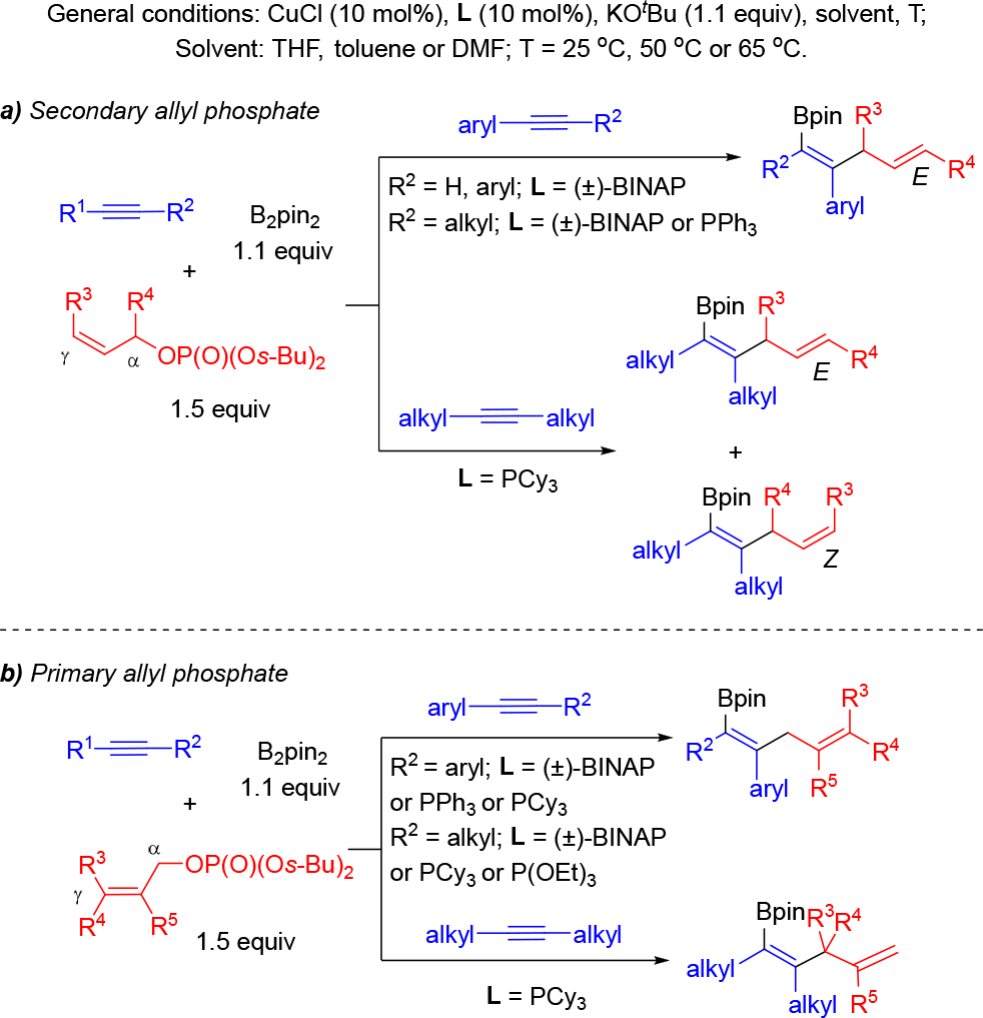
Copper(I)-Catalyzed Allylboration of Alkynes with Allylic Phosphates

Soon thereafter, Zhong and co-workers demonstrated that this selectivity trend is not general when applied to other alkynes and allylic phosphates under copper/phosphine catalysis.^15^ Reaction conditions (ligand, solvent, and temperature) had to be optimized for every alkyne substitution pattern. Importantly, the regioselectivity of the allylic substitution step operates under substrate control. Secondary allylic phosphates in combination with phenylacetylene derivatives and internal diaryl and alkyl(aryl)alkynes led to the formation of the γ-allylation product using (±)-BINAP or PPh_3_ as ligand. In combination with internal dialkyl alkynes, these allylic substrates led to mixtures of regioisomers using PCy_3_ as the ligand (Scheme 3a). Under these conditions, the α-substitution pathway led to the formation of the *Z*-product. For primary allylic phosphates, the reaction with internal diaryl alkynes provided the α-allylation isomer as the major product (Scheme 3b). In contrast, the use of dialkylalkynes led preferentially to the γ-allylation isomer while internal alkyl(aryl)alkynes furnished regioisomeric mixtures.

**Scheme 4:**
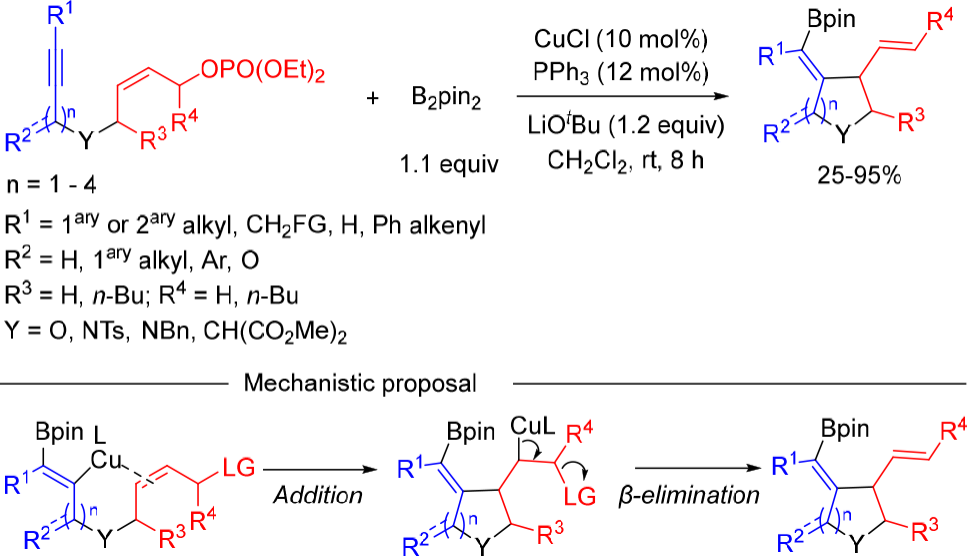
Intramolecular Copper-Catalyzed Borylative Cyclization of 1,*n*-Enynyl Phosphates

An intramolecular version of this transformation was reported by Bai and Zhu et al.^16^ By using Cu/PPh_3_ catalysis, a series of (*Z*)-enynyl phosphates were converted into different-sized heterocycles bearing an exocyclic alkenyl boronate (Scheme 4). A mechanism involving an addition–elimination pathway was proposed for the allylic substitution step that occurs with γ-allylation selectivity likely because of the intramolecular nature of the transformation.

**Scheme 5:**
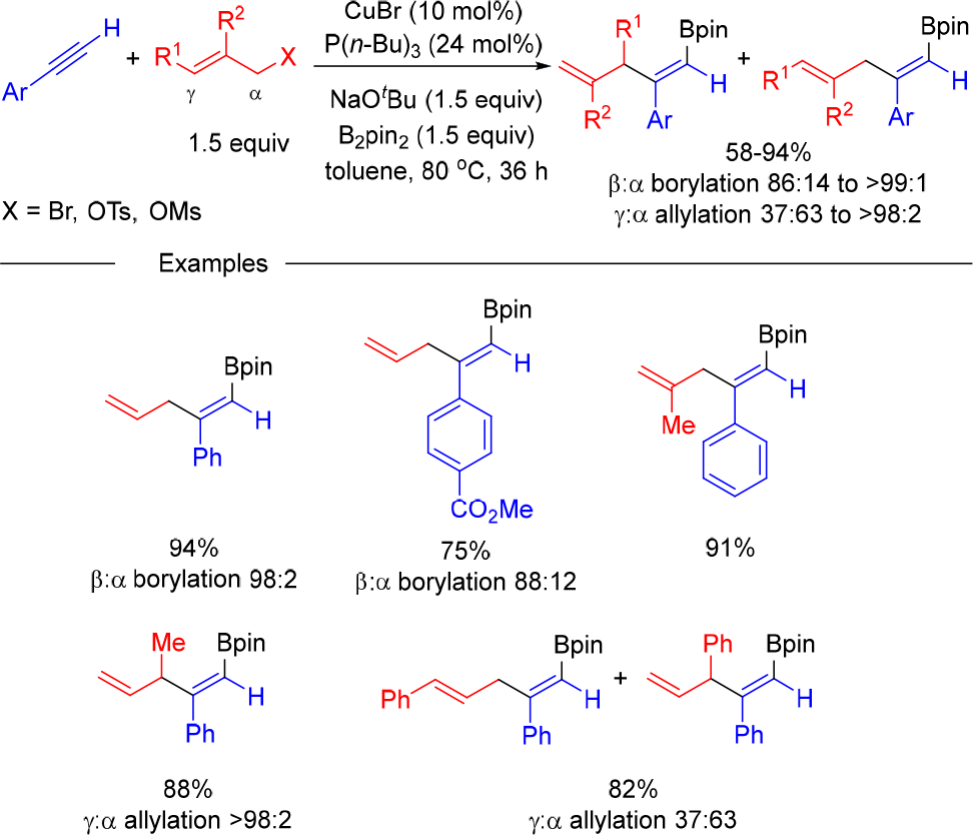
Copper-Catalyzed Allylboration of Alkynes with Allylic Bromides

Copper/phosphine catalysis was also extended to the allylboration of alkynes with allyl (pseudo)halides. The group of Wang reported that the use of CuBr and P(*n*-Bu)_3_ as catalyst can promote the three-component reaction to afford skipped dienyl boronates in good yield.^17^ However, the reaction is limited to terminal aryl alkynes, and variable mixtures of α- and γ-allylation isomers are obtained when 1,3-disubstituted allyl (pseudo)halides are used (Scheme 5).

**Scheme 6:**
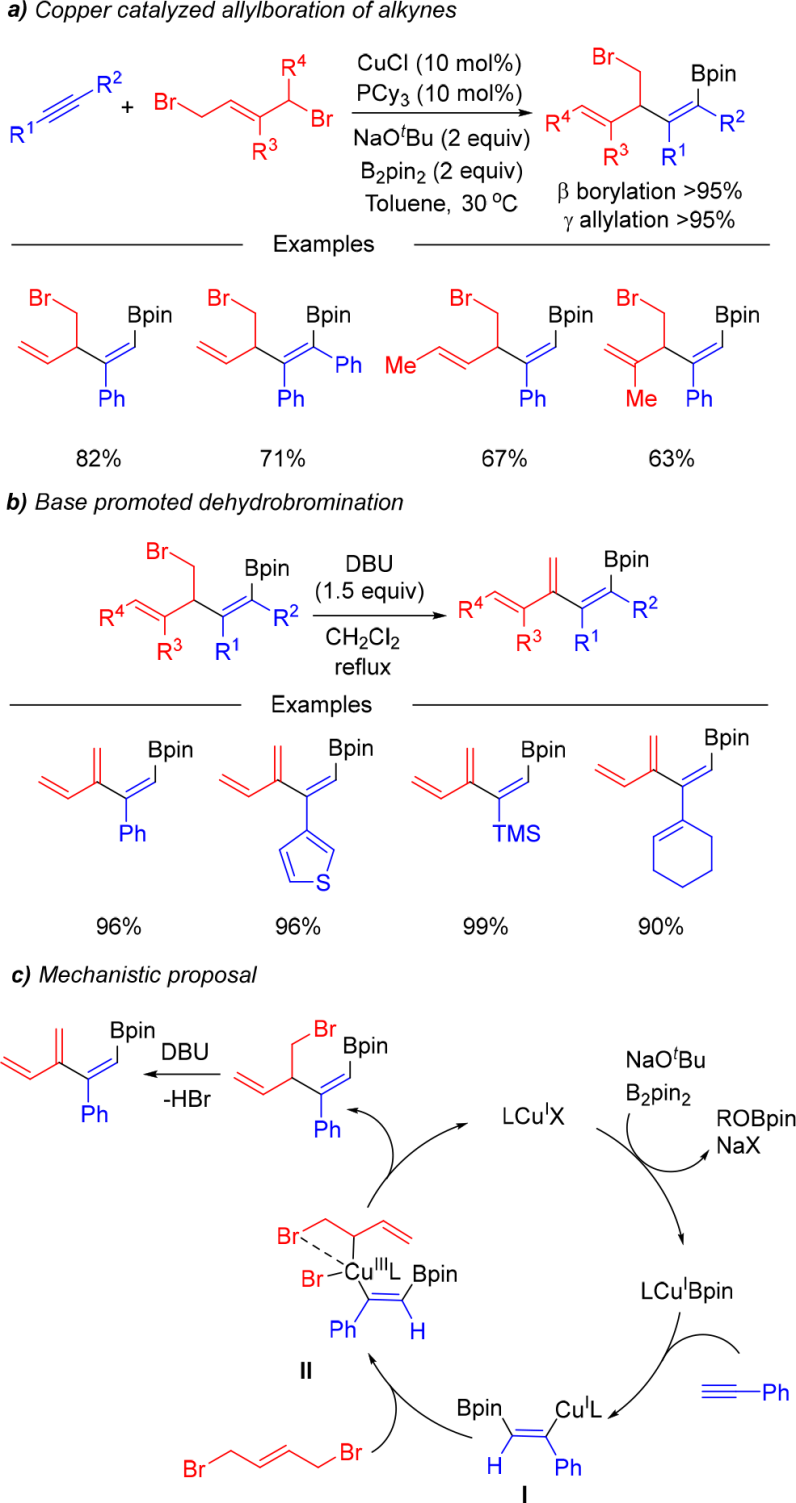
Copper-Catalyzed Borylative Coupling of Alkynes and 1,4-Dibromo-2-butenes

In 2018, our group described the copper-catalyzed allylboration of alkynes using 1,4-dibromo-2-butenes as starting materials and CuCl/PCy_3_ as catalyst (Scheme 6).^18^ The reaction tolerated terminal and internal alkynes and provided bromo-substituted borylated skipped dienes with high levels of chemo-, regio-, and stereoselectivity. The enhanced reactivity, as well as the excellent γ-allylation selectivity, is likely due to the stabilization of the Cu^III^ intermediate **II** by intramolecular coordination of the remaining bromide to the Cu atom.

Interestingly, the bromo-substituted borylated skipped dienes could be easily converted to the corresponding borylated dendralenes by a base-promoted dehydrobromination. This new type of organoboron compounds proved to be very versatile reagents, as illustrated with a range of novel synthetic transformations.^19^

**Scheme 7:**
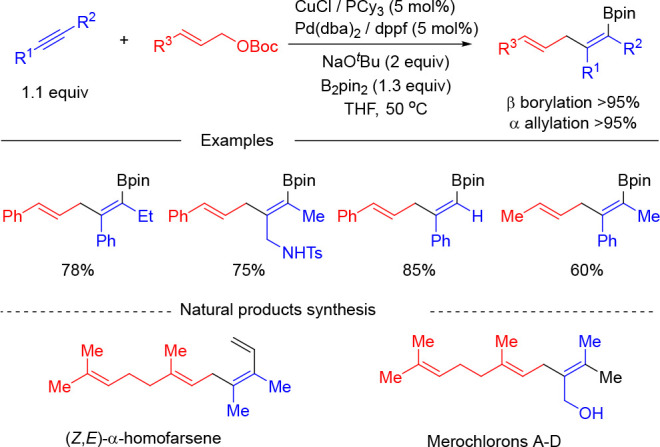
Synergistic Copper/Palladium-Catalyzed Borylative Coupling of Alkynes and Allylic Carbonates

With the aim to seek an alkyne allylboration methodology in which regioselectivity operates under catalyst control, our group focused on a strategy based on the synergistic combination of Cu and Pd catalysis. We envisaged a strategy on the basis of the catalytic generation of a β-borylated alkenylcopper intermediate that undergoes Pd-catalyzed allylic substitution in a process where the regioselectivity of the allylic substitution step is controlled by the Pd catalyst. The evaluation of different combinations of Cu and Pd catalysts led to the finding that the catalytic system comprising CuCl/PCy_3_ and Pd(dba)_2_/dppf was optimal to render the allylboration regioselective (β-borylation > 95% and α-allylation > 95%) (Scheme 7).^20^ The reaction tolerates terminal and internal alkynes bearing both aliphatic and (hetero)aromatic substituents, which can be efficiently reacted with primary and secondary allylic carbonates. The method could also be extended to the coupling of trisubstituted allylic carbonates, such as geraniol or nerol derivatives. The synthetic utility of the methodology was demonstrated by the total synthesis of (*Z*,*E*)-α-homofarnesene, a trial pheromone of the fire ant, and isosesquilavandulyl alcohol, a key intermediate in the synthesis of merochlorins A–D that are marine meroterpenoid secondary metabolites with potent antibiotic activity.

**Scheme 8:**
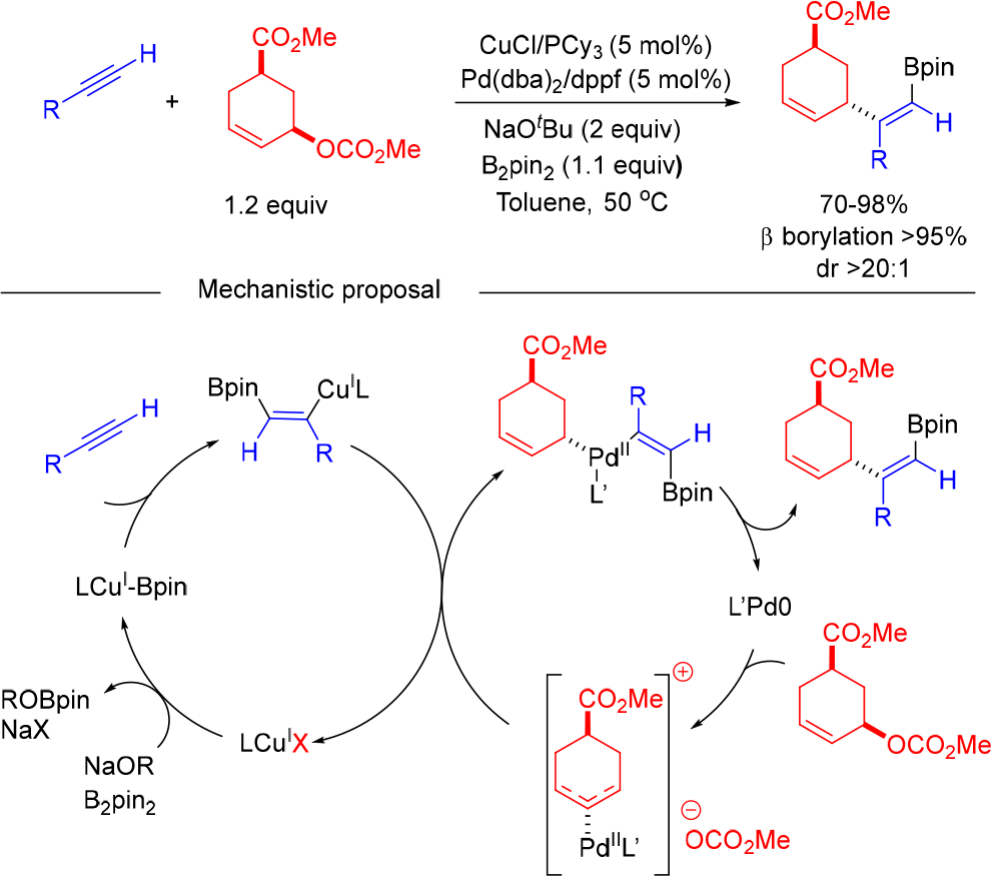
Alkyne Allylboration Reaction with Diastereomerically Pure *cis*-Cyclic Allyl Carbonates and Mechanistic Proposal

Mechanistic studies confirmed that both catalytic cycles are likely connected by a transmetalation step. More specifically, total inversion of configuration was observed when diastereomerically pure allyl carbonates were reacted with different alkynes and B_2_pin_2_ (Scheme 8).^21^ Excellent diastereocontrol was observed, with *cis*-allylcarbonates providing the corresponding products as single *trans*-diastereomers in good yield with excellent chemo- and regioselectivity. Similarly, a pure *trans*-cyclic allyl chloride led to the exclusive formation of the *cis* product when reacted with 3-ethynylthiophene and B_2_pin_2_ under our optimized Cu/Pd catalytic conditions. These results clearly suggest that reaction occurs via stereoinvertive oxidative addition of the allyl substrate to the Pd(0) catalyst, followed by inner-sphere transmetalation of the alkenylcopper intermediate to the Pd center (likely after π- to σ-allyl isomerization) and final stereoretentive reductive elimination.

**Scheme 9:**
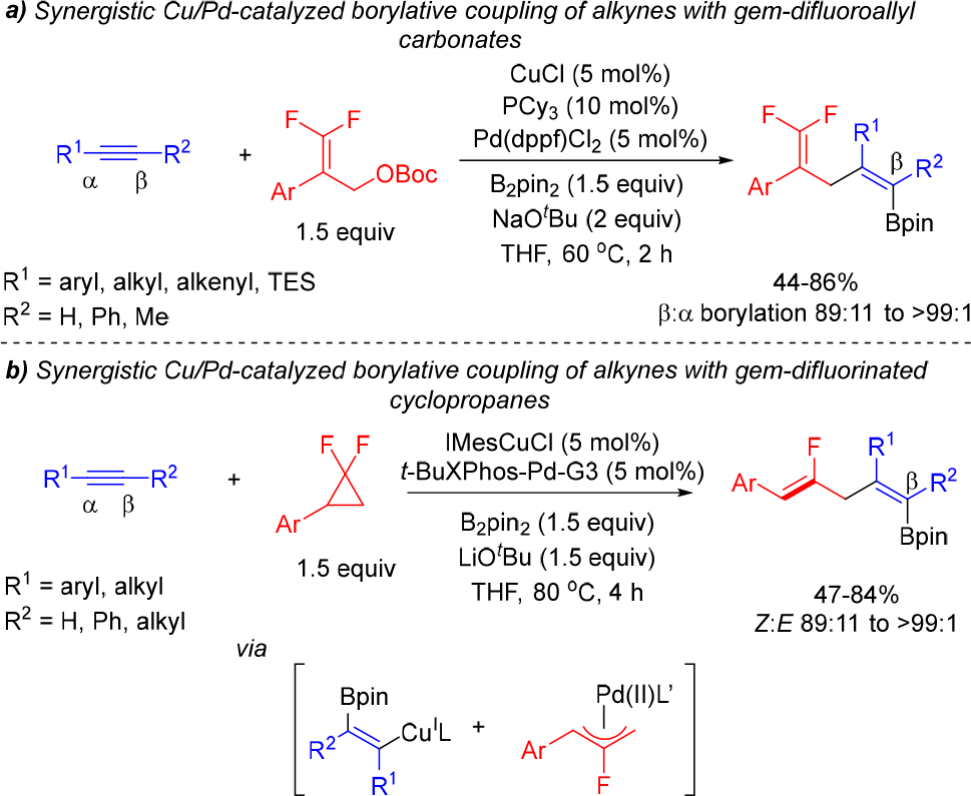
Synergistic Cu/Pd-Catalyzed Borylative Coupling of Alkynes with (a) *gem*-Difluoroallyl Carbonates and (b) *gem*-Difluorinated Cyclopropanes

After our initial studies on the Cu/Pd-catalyzed allylboration of alkynes, Gong, Fu and co-workers reported a similar methodology for the borylative coupling of alkynes with *gem*-difluoroallyl carbonates (Scheme 9a).^22^ The method provided access to *gem*-difluoro borylated 1,4-dienes with a great functional group tolerance and a broad scope of alkynes, although it was limited to 2-aryl-substituted *gem*-difluoroallyl carbonates.

Following on their studies, Gong and Fu et al. demonstrated the merge of the Pd-catalyzed C–C bond activation/C–F bond cleavage of *gem*-difluorinated cyclopropanes with the Cu-catalyzed alkyne borylcupration to develop a Cu/Pd-catalyzed *cis*-borylfluoroallylation of alkynes (Scheme 9b).^23^ In this transformation, a Pd/*t*-Bu-XPhos catalyst promotes the *gem*-difluorinated cyclopropane ring opening to generate an allylpalladium(II) complex that reacts with the alkenylcopper intermediate to furnish a monofluorinated borylated 1,4-diene.

**Scheme 10:**
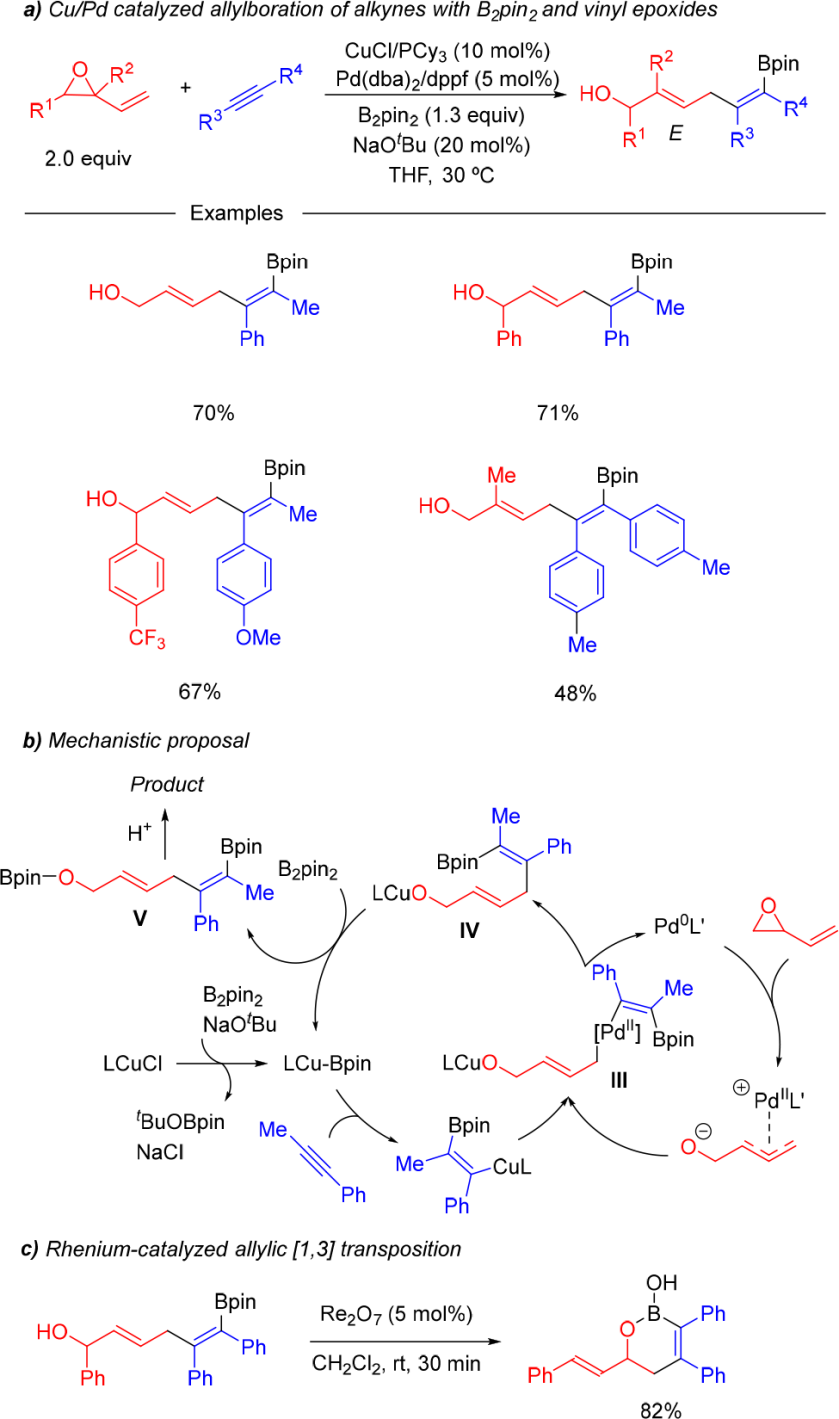
Synergistic Cu/Pd-Catalyzed Borylative Coupling of Alkynes and Vinyl Epoxides

Synergistic Cu/Pd-catalyzed alkyne allylboration was extended to the use of vinyl epoxides by our group (Scheme 10).^24^ Substitution reactions with this type of substrate entail an important challenge in terms of control over the regioselectivity (1,2- vs 1,4-addition) and the stereoselectivity of the resulting allylic alcohol. The catalytic system comprising CuCl/PCy_3_ and Pd(dba)_2_/dppf proved to be efficient for the three-component coupling between alkynes, B_2_pin_2_, and vinyl epoxides and provided bifunctional skipped dienes as single 1,4-addition products featuring a (*Z*)-alkenyl boronate and an (*E*)-allylic alcohol. Besides the regio- and stereoselectivity, control over the competing direct addition of B_2_pin_2_ to the vinyl epoxide represented a major issue in this transformation. In this sense, the use of a 2:1 Cu/Pd ratio and slow addition of the vinyl epoxide were key to achieving total chemoselectivity control. Interestingly, the reaction could be carried out with a catalytic amount of NaO^*t*^Bu. This is likely due to the ability of copper alkoxide **IV**, which originates from the reductive elimination in bimetallic intermediate **III**, to react with B_2_pin_2_ to regenerate the active Cu-Bpin species (Scheme 10b). In terms of synthetic versatility, the orthogonal functionalization of these products makes them versatile building blocks, as illustrated by their rhenium-catalyzed conversion into cyclic boronic acids (Scheme 10c), a structural motif that has recently gained interest in the pharmaceutical industry.

## Regio- and Enantioselective Alkyne Allylboration

**Scheme 11:**
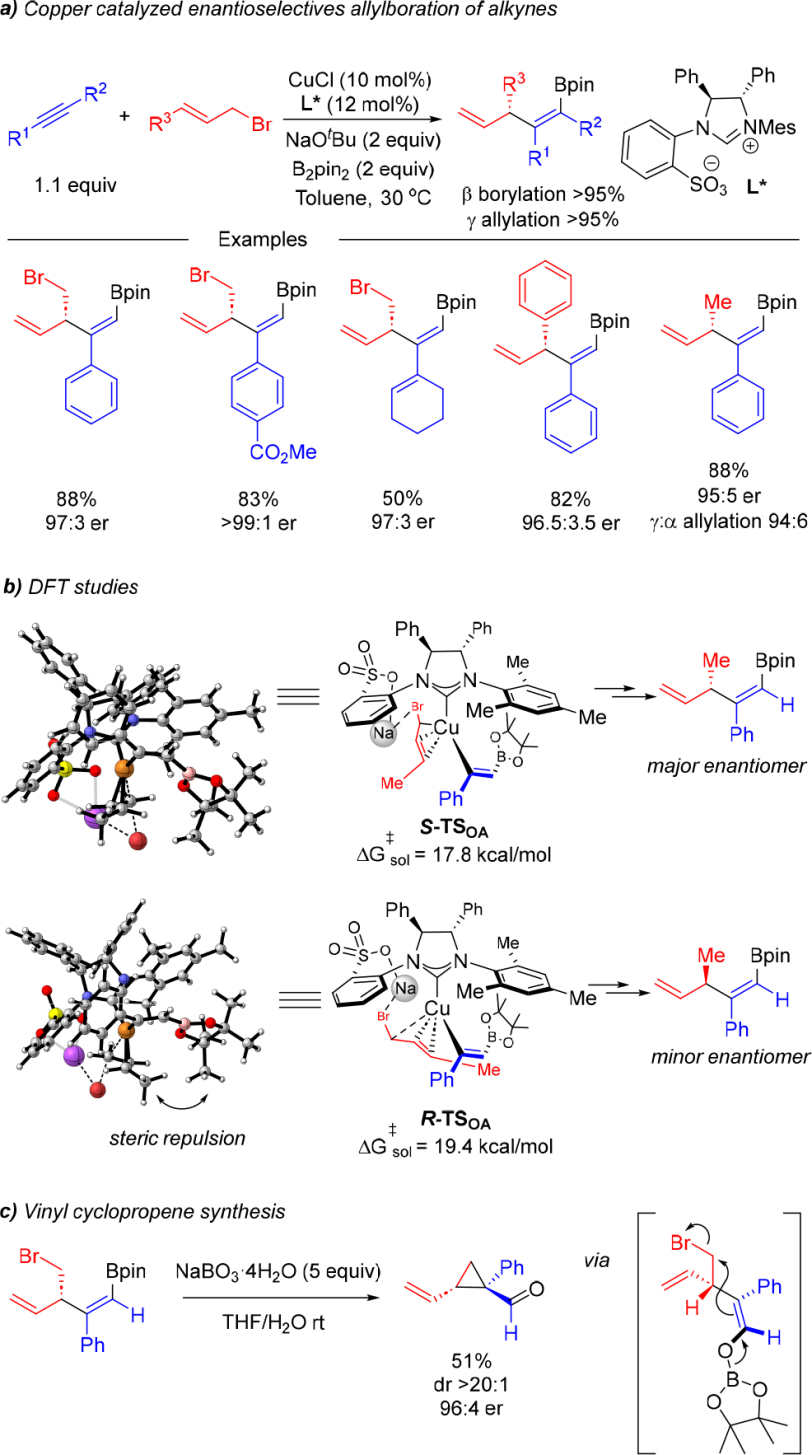
Copper-Catalyzed Asymmetric Allylboration of Alkynes with Allylic Bromides

In 2019, our group developed the first copper-catalyzed asymmetric allylboration of alkynes (Scheme 11).^25^ The reaction involves the coupling of a terminal alkyne, an allylic bromide, and B_2_pin_2_ and provides chiral-branched borylated 1,4-dienes with very good chemo-, stereo-, regio-, and enantioselectivity. Importantly, the reaction could be applied to a wide range of allylic bromides, including 1,4-dibromo-2-butene, crotyl bromide, or different cinnamyl bromide derivatives. The use of a chiral sulfonate-bearing NHC ligand was key to achieving these high levels of selectivity. DFT calculations supported the presence of a metal cation bridge interaction between the sulfonate unit of the ligand and the bromide that is essential to orientate and activate the allylic substrate and to provide a conformationally rigid transition-state structure that facilitates enantioface differentiation in the enantiodetermining oxidative addition step. On the basis of this stereochemical model, it was proposed that the energy difference between both enantiomeric transition states mainly lies in the repulsive interaction between the substituent of the allylic substrate with the Bpin unit that is engendered in the structure leading to the minor enantiomer (Scheme 11b).

An attractive feature of this method is the high degree of functionalization that can be achieved in a chiral molecule that can be obtained from simple starting materials. The synthetic utility of these molecules was illustrated via an oxidative cyclopropanation reaction that provides a vinylcyclopropane carbaldehyde in which a new all-carbon quaternary center is generated with high diastereoselectivity (Scheme 11c).

## Regio-, Enantio-, and *Z*-Selective Alkyne Allylboration

**Scheme 12:**
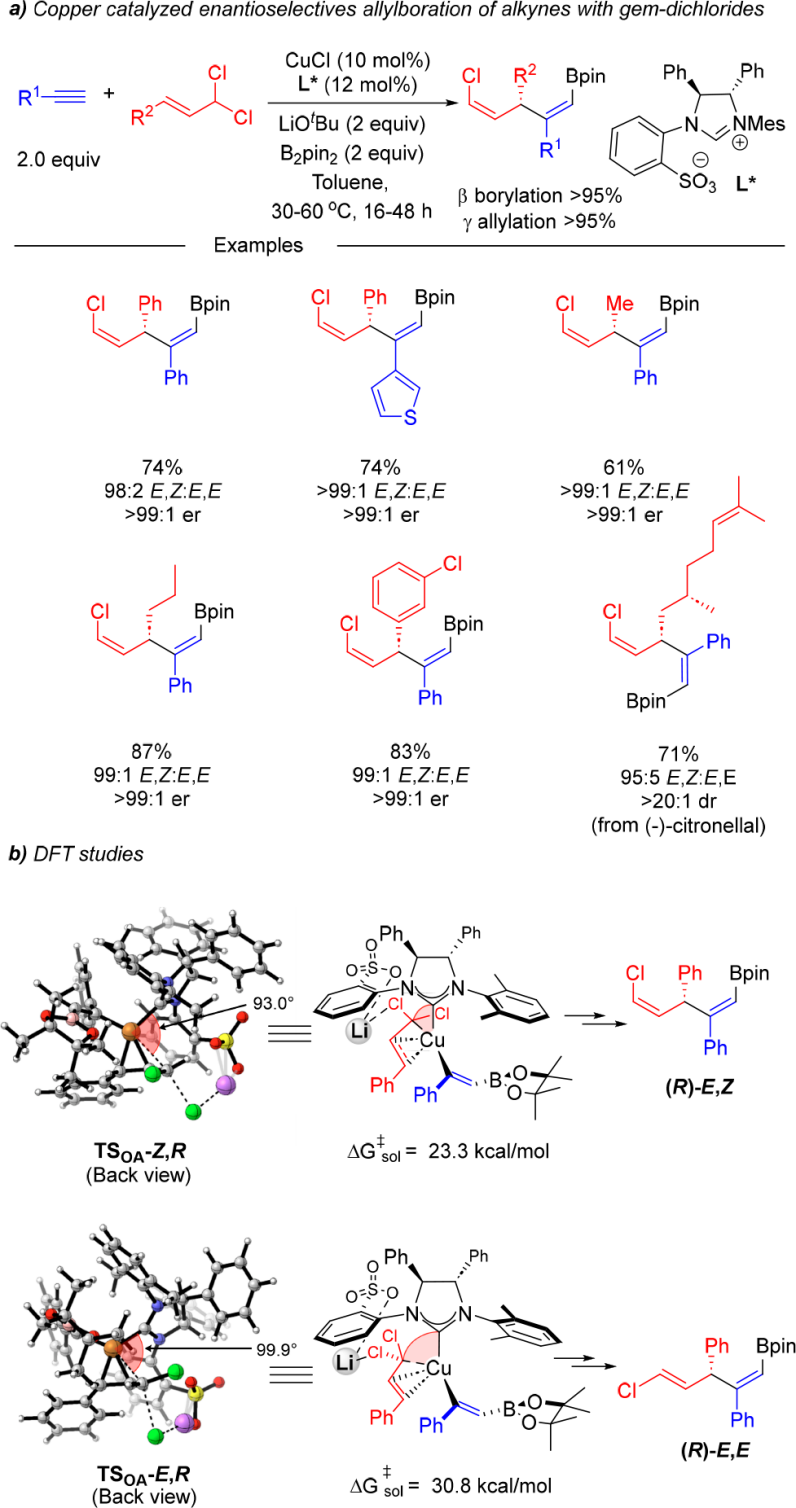
Copper-Catalyzed Asymmetric Allylboration of Alkynes with Secondary Allylic *gem*-Dichlorides

Following on our studies on catalytic asymmetric borylative couplings, in 2022 we reported a copper-catalyzed enantio- and diastereoselective allylboration of alkynes with allylic *gem*-dichlorides (Scheme 12).^26^ The use of a secondary allylic substrate, such an allylic *gem*-dichloride, entails an extra selectivity challenge since the presence of two chlorine atoms in the allylic substrate results in an additional stereocontrol element, i.e., the diastereoselective formation of a 1,2-disubstituted alkenyl chloride. By using a copper catalyst comprising a chiral sulfonate-bearing NHC ligand, a range of terminal alkynes bearing (hetero)aromatic and aliphatic substituents could be coupled with both aliphatic and aromatic allylic *gem*-dichlorides and B_2_pin_2_ to provide the corresponding bifunctional skipped dienes bearing an alkenyl boronate and an alkenyl chloride with perfect regiocontrol (β-borylation and γ-allylation > 95%) and excellent enantio- and *E*,*Z*-selectivity. Additionally, the use of chiral substrates with a preinstalled stereocenter was also demonstrated with the newly generated stereocenter being created with total diastereoselectivity.

Mechanistic studies revealed that the cation-bridged interaction that is established between the ligand and the allylic *gem*-dichloride is crucial to controlling not only the enantioselectivity but also the *Z*-selectivity observed in the formation of the alkenyl chloride (Scheme 12b). The origins of enantioselectivity were similar to those observed in the asymmetric allylboration of alkynes with allylic bromides.^25^ Regarding the *Z*-selectivity, the lithium cation bridge imposes a bigger C_NHC_–Cu–Cα angle in the transition state leading to the (*E*)-alkenyl chloride that pushes the allyl substrate closer to the Bpin unit, thereby leading to a larger repulsive interaction. The size of the metal cation was crucial in the control of the *Z*-selectivity, as shown with the decreased selectivity observed when NaO^*t*^Bu was used instead of LiO^*t*^Bu (*E*,*Z*/*E*,*E* 84:16 vs 99:1). Distortion–interaction analysis of the transition states in both systems suggested that this lower selectivity may be due to a higher stabilizing interaction present in the sodium-based transition state leading to the minor *E*-isomer that likely arises from the larger size of the Na cation, which might facilitate a more effective interaction with both chlorine atoms.

## Alkyne Borylcupration Regioselectivity: α- versus β-Borylation

**Scheme 13:**
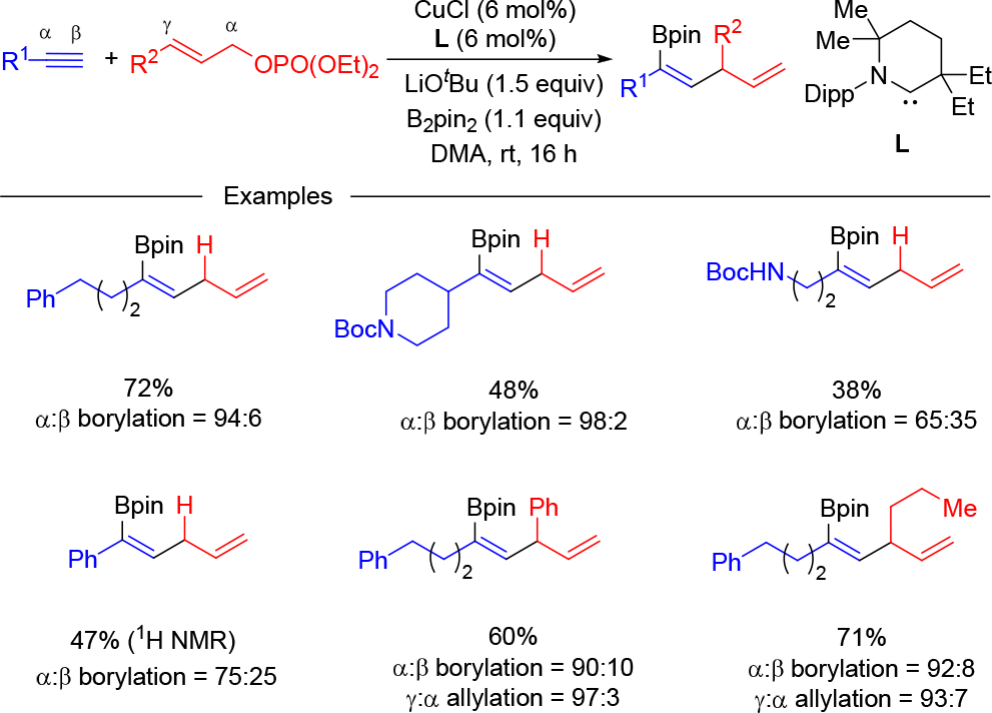
Copper-Catalyzed α-Selective Allylboration of Terminal Alkynes

Another point of selectivity inherent to alkyne borylative couplings is control over the regioselectivity in the addition of the boryl–copper complex to the alkyne when this has an unsymmetrical structure. This issue is not trivial and is usually dictated by an interplay between the nature of the substituent(s) on the alkyne, the steric and electronic properties of the Cu/ligand catalyst, and the identity of the diboron compound.^27−29^ All of the catalytic systems described above on the basis of the Cu/phosphine or Cu/NHC complexes deliver the boryl group to the terminal β position when applied to terminal alkynes with good to excellent selectivity. Examples involving internal unsymmetrical alkynes have been associated with the use of aryl alkyl alkynes or propargylic internal alkynes where the boryl group adds to the more electropositive position of the alkyne, thus also providing β-borylation products. Recently, Engle and co-workers described how the use of strongly σ-donating cyclic(alkyl)(amino)carbene (CAAC) ligands can override substituent effects allowing for α-selective borylation of terminal alkynes.^30^ This strategy has been used by the same authors to develop a copper-catalyzed allylboration of terminal alkynes that provides selective access to borylated skipped dienes bearing an internal alkenylboronate (Scheme 13).^31^ The methodology could be efficiently applied to the coupling of terminal aliphatic alkynes with allylic diethylphosphates and B_2_pin_2_ by using LiO^*t*^Bu as base. A decrease in the α-selectivity was observed with alkynes bearing an aliphatic substituent with a close electronegative group, as well as when phenylacetylene was used. Allylic phosphates with substitution at the gamma position proved also to be efficient for this transformation and afforded the corresponding products with high levels of regioselecivity (>90% α-borylation and γ-allylation).

## Conclusions

As outlined above, the area of alkyne allylboration has grown significantly in the last years. Several methodologies in which the different selectivity points can be controlled either by substrate selection or by catalyst design have been reported. Moreover, divergent selectivity outcomes can be achieved by switching from copper to copper/palladium catalysis. Despite these advances, several challenges still must be met. Enantioselective Cu/Pd-catalyzed alkyne allylboration has only been demonstrated with moderate enantioselectivity.^21^ Additional efforts should be devoted toward the identification of a catalytic system, on the basis of either Cu/Pd or another bimetallic combination, capable of rendering a fully enantioselective alkyne allylboration involving, for instance, the use of a secondary allylic substrate with α-allylation selectivity. Within this context, the use of racemic allylic substrates in asymmetric carboboration of unsaturated hydrocarbons represents an almost uncharted territory, and thus, the development of enantioconvergent allylboration processes would be very valuable. Desymmetrization of *meso* compounds involving an allylboration strategy would also contribute to significantly expand the chemical space of these transformations. Another important issue to be solved is the identification of a system in which the Cu catalyst can promote α-selective borylation of the alkyne while undergoing an enantioselective allylic substitution step. Perhaps the use of different diboron reagents with attenuated Lewis acidity^27^ or chiral CAAC ligands may be a good basis to achieve this goal. Another missing spot is the development of an enantioselective intramolecular allylboration reaction. Finally, the alkyne scope should also be expanded. While both terminal and internal alkynes have proven efficient for several allylboration reactions, enantioselective transformations have been mainly applied to aromatic and aliphatic terminal alkynes, the latter being challenging in some cases. More work needs to be done to extend these systems to internal alkynes or even to the simplest acetylene. Advances in this area will continue to help meet the challenge of the synthesis of complex organic molecules through the selective transformation of simple hydrocarbons.
